# Energy-Efficient Swarming Flight Formation Transitions Using the Improved Fair Hungarian Algorithm

**DOI:** 10.3390/s21041260

**Published:** 2021-02-10

**Authors:** SungTae Moon, Donghun Lee, Dongoo Lee, Doyoon Kim, Hyochoong Bang

**Affiliations:** 1 Aerospace Systems and Control Laboratory, Korea Advanced Institute of Science and Technology (KAIST), Deajon 34141, Korea; stmoon@kari.re.kr; 2 Korea Aerospace Research Institute (KARI), Deajon 34133, Korea; ldg810@kari.re.kr (D.L.); doyoonkim@kari.re.kr (D.K.); 3 School of Aerospace and Mechanical Engineering, Korea Aerospace University (KAU), Goyang-si 10540, Korea; donghlee@kau.ac.kr

**Keywords:** swarming flight system, Hungarian algorithm, RTK-GPS

## Abstract

Recently, drone shows have impressed many people through a convergence of technology and art. However, these demonstrations have limited operating hours based on the battery life. Thus, it is important to minimize the unnecessary transition time between scenes without collision to increase operating time. This paper proposes a fast and energy-efficient scene transition algorithm that minimizes the transition times between scenes. This algorithm reduces the maximum drone movement distance to increase the operating time and exploits a multilayer method to avoid collisions between drones. In addition, a swarming flight system including robust communication and position estimation is presented as a concrete experimental system. The proposed algorithm was verified using the swarming flight system at a drone show performed with 100 drones.

## 1. Introduction

As interest in unmanned aerial vehicles (UAVs) increases, many applications utilizing UAVs have been developed in various fields, such as delivery services [1] and reconnaissance. In particular, drone shows impressed many people around the world through a convergence of technology and art [2]. These demonstrations have shown significant advances in swarming flight technology. 

For the swarming flight, various studies have been conducted in real environments with simulation [3,4,5]. In the early stages of research on swarming flight in a real environment, most researches were conducted indoors [6,7]. In the case of indoor swarming flight, motion capture or images can be used for position estimation. With the development of various sensors, various teams attempted to demonstrate swarm flight outdoors [8,9,10]. Later, Intel presented the first successful demonstration with 100 drones, in collaboration with Ars Electronica [10], and later presented a drone show involving more than 1000 drones during the 2018 Pyeongchang Winter Olympics, which was published in the Guinness Book of World Records. However, the system including communication and scenario generation has not been revealed because the system was developed privately.

In addition, the drone shows have limited operating hours based on the battery life. Thus, it is important to minimize the unnecessary transition time between scenes without collision to increase operating time. This transition problem can be regarded as a classical linear sum assignment problem (LSAP). The LSAP is one of the most famous problems in combinatorial optimization. The objective in this problem is to optimally assign agents to tasks individually such that the minimum cost or maximum profit can be obtained.

To solve the LSAP, diverse algorithms have been developed, including the simplex algorithm (standard linear programming) [11,12,13], the Hungarian algorithm [14,15], a neural algorithm [16], and the auction algorithm [17]. Each algorithm has undergone further development to improve its time complexity [18]. Among the various LSAP algorithms, the Hungarian algorithm is the well-known optimal solution.

Many researchers have performed studies on the LSAP based on the Hungarian algorithm for the task assignment of multiple robots such as drones. Smriti et al. proposed a distributed version of the Hungarian method to improve task assignment efficiency [19]. Xiangming et al. developed an intelligent target detection method of UAV swarms using the Hungarian algorithm-based path planning method and three dimensions (3D) real-time probability map which estimates the possibility of detecting new targets [20]. Amir et al. described the minimum cost drone-station matching algorithm considering energy and distance for a smart city [21]. Sarah et al. proposed the Decentralized Hungarian-Based Algorithm (DHBA) to improve computational time and the optimality of assignments [22]. 

As shown in Table 1, most algorithms based on the Hungarian method have attempted to find an optimal solution that minimizes or maximizes the weight or improves the time complexity. However, although these algorithms can find an optimal solution that minimizes the sum of the transition times for each drone, it does not guarantee the minimization of the transition time in the 7drone shows, as the transition is completed only after all the drones have completely moved to the next scene position. Even when most drones have arrived at their destinations, these drones must wait until the last drone arrives because all the drones must be deployed in their designated positions for the next scene. As a result, the Hungarian algorithm is not efficient in terms of drone battery usage, and consequently, the overall operating time is reduced.

In this paper, a Fair Hungarian algorithm is proposed to generate a scenario that includes transitions between scenes within a short time without collision. The goal of the proposed algorithm is to achieve fast and energy-efficient scene transitions while minimizing the transition times between scenes during the generation of a scenario.

The main contributions of this paper can be summarized as follows:

(1) To increase the operating time for drone shows, the Fair Hungarian algorithm is proposed to achieve fair energy consumption. The proposed algorithm equalizes the energy demand of the drones by minimizing the maximum movement distance between drones in a swarming flight scenario.
(2) The drone show technology stacked on the veil is discussed. In this paper, methods to realize efficient communication and reliable position estimation for a swarming flight system are discussed. The communication mechanism can operate regardless of the number of drones. The position estimation based on the real time kinematic global positioning system (RTK-GPS) can switch mode smoothly when the RTK-GPS is not used.
(3) The algorithm and system are verified through implementation in drone shows involving 100 drones with numerical experiments.

The paper is organized as follows. Section 2 describes the overall architecture of the swarming flight system considering efficient communication and position estimation. Section 3 discusses how to generate a safe and efficient scenario. Section 4 discusses the results of swarming flight experiments, and finally, Section 5 presents the conclusions and future research plans.

## 2. System Architecture

### 2.1. Overall Architecture

**Figure 1:**
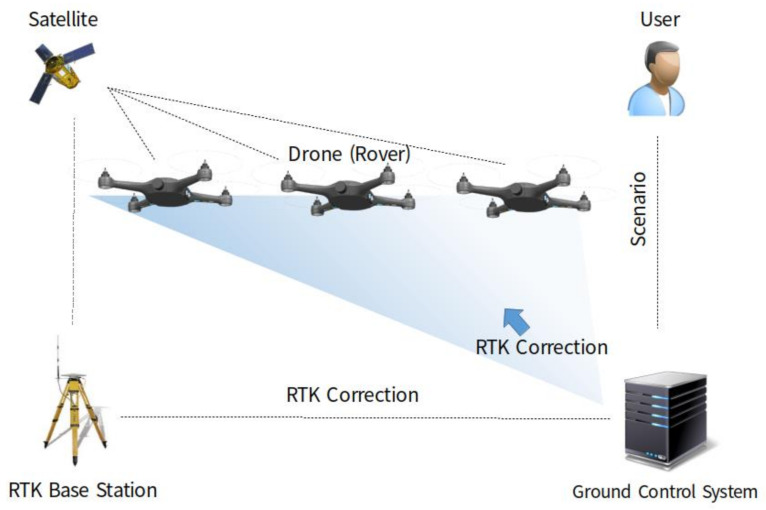
Deployment of the proposed swarming flight system.

A swarming flight system should be deployed as simply as possible to facilitate the operation of a large number of drones and frequent movements between show locations [23]. Therefore, the proposed swarming flight system is designed to require only a simple GCS to ensure convenience of operation. The swarming flight system consists of a GCS, an RTK-GPS base station, drones, and scenarios generated by the user, as shown in Figure 1. The proposed GCS can be implemented on a laptop computer because it does not require high performance equipment for command transmission. The base station is used to transmit the correction data to enable precise position estimation based on RTK-GPS sensors, which are more precise than global positioning system (GPS) sensors [24]. Using this system, position estimation at the centimeter level can be realized. Each drone is equipped with a PX4 flight control computer (FCC) [25,26] including various algorithms using position control, motion equations and electromagnetic theory [27,28,29] based on open-source software. Notably, the PX4 FCC [30] is modified to enable efficient communication as well as robust control and position estimation during the swarming flight. The scenario for a drone show is prepared by the user before the flight and stored in the drones. Finally, each drone communicates with the GCS using the micro air vehicle link (MAVLink) [31], which is a lightweight messaging protocol for communication via Wi-Fi.

In an emergency situation, the GCS can detect and control each individual malfunctioning drone during the scenario-based flight. However, if the communication system of the GCS experiences problems and all communication is lost, the GCS cannot control the drones. In this case, all the drones can be controlled by a grouped remote controller and landed in the direct downward direction to protect spectators.

**Figure 2:**
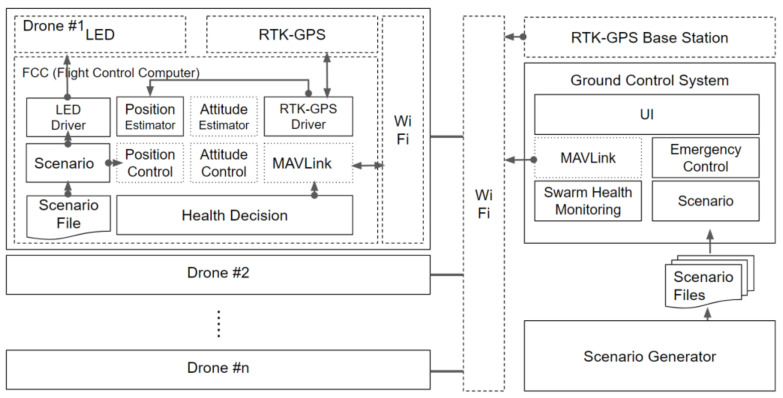
Control architecture for swarming flight (solid box: contributed software component, dotted box: PX4 software component, dashed box: hardware module).

The main components of the drones and GCS are shown in Figure 2. The drone system consists of an RTK-GPS module, a Wi-Fi module, a light emitting diode (LED), and an FCC module. In the FCC, several components are added to the basic PX4 FCC module. Each component communicates with other components through the message-driven method of a micro object request broker (µORB) [26]. The µORB is designed following a publish-subscribe model. The MAVLink component manages the communication between the GCS and drone by using the MAVLink protocol, which is optimized for the drone. The scenario component is operated in accordance with an already-generated scenario file, which is transmitted from the GCS. This component passes the position and LED commands to the position controller and LED component, respectively. Using a mode switching algorithm mentioned in Section 2.3, the position estimator component can robustly estimate the drone’s position even if the fixed mode of the RTK-GPS module suddenly fails. The monitoring component mentioned in Section 2.2 checks the health of the drone and operates within the drone to reduce the information exchanged with the GCS.

The ground system consists of the RTK-GPS base station, the GCS, and the scenario creator. The RTK-GPS base station transmits the RTK-GPS correction information to each drone. The GCS has several components. The MAVLink component is the same as the corresponding component on each drone. To check the status of each drone in real time, the swarm monitoring component monitors the status of each drone based on the information received from the drone. The scenario component transmits the scenario information provided by the scenario generator mentioned in Section 3 to each drone. The emergency control component controls the drones that experience problems during the scenario flight.

### 2.2. Efficient Communication

As the number of drones involved in swarming flight increases, communication limitations due to the bandwidth are encountered. In addition, although wireless routers and other access points can theoretically support up to approximately 250 connected devices, such a large number of drones cannot be simultaneously connected because radio interference among the Wi-Fi devices may deteriorate the network performance due to frequent rebroadcasting of the messages that fail to reach their destinations, eventually leading to connection drops [32].

Nevertheless, it is essential to continuously transmit the correction data to maintain the fixed RTK-GPS mode to enable precise position estimation during the swarming flight. If the RTK-GPS correction data stream stops for a long time due to burst packet loss caused by unexpected network congestion, the fixed mode will change to the floating mode. Therefore, it is important to maintain communication stability and to protect against burst packet loss to maintain the fixed RTK-GPS mode.

**Figure 3:**
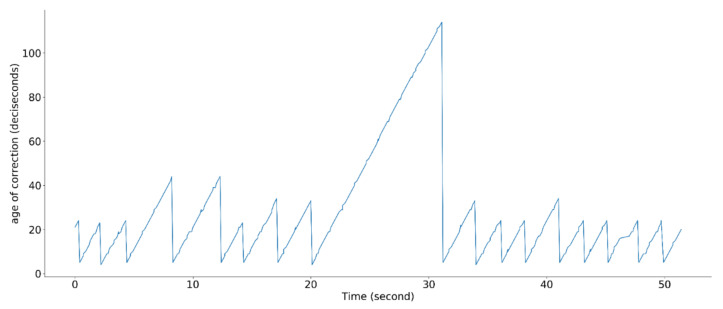
Age-of-correction status.

Fortunately, the fixed mode can be maintained for a limited time even if the transmission of the RTK-GPS correction data stops. To check the health status of the fixed mode, the age of the correction information is used, as shown in Figure 3. When a drone receives the correction data, the age of the correction information is reset.

Various communication algorithms have been proposed in order to stably operate many drones at the same time [33,34]. However, such network systems cannot incorporate all drones for a large swarming flight system because of the limitations of Wi-Fi technology. In the Wi-Fi framework, the carrier sense multiple access with collision avoidance (CSMA/CA) mechanism is used to manage the collision of transmitted packets [35]. This can be used to manage undefined nodes that are accessed randomly. However, this mechanism cannot support reliable real-time communication because the throughput of Wi-Fi decreases dramatically when many drones are simultaneously connected [36]. To solve this problem, the amount of data to be exchanged in the proposed system is reduced as much as possible by removing unnecessary information. This approach allows for an unlimited number of drones to be operated using the proposed system.

**Figure 4:**
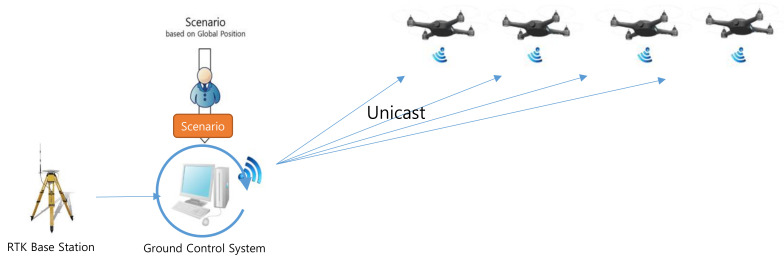
Traditional communication approach for the swarming flight.

To reduce the amount of data exchanged in the proposed system, the data transmitted from the GCS to the drones should be optimized. Normally, GCS sends a command to the drone, and the drone sends the drone’s status to the GCS. To compare the performance, we assume the same environment. The GCS transmits the correction for RTK-GPS and scenario at 1 and 10 Hz. The drones transmit the drone’s status at 1 Hz. In the traditional swarming flight system which is depicted in Figure 4, the communication traffic ($d_{s1}$) increases linearly with the number of drones, as follows:

(1) $$d_{s1}=n\times (d_{base}\times 1+d_{scen}\times 10+d_{status}\times 1)$$

where $d_{base}$ and $d_{scen}$ denote the amounts of correction data and scenario data, respectively, transmitted from the GCS to a drone. $d_{status}$ denotes the amount of status data, such as battery data, transmitted from a drone to the GCS and n is the number of drones. This approach is useful for unknown missions such as collision avoidance in response to some event, because the drones can be controlled in real time. However, it is not suitable for drone shows that require a certain amount of traffic regardless of the number of drones.

**Figure 5:**
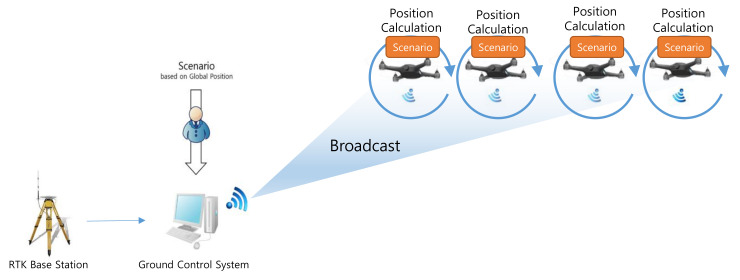
Proposed communication approach for the swarming flight.

In the proposed system, to reduce the communication traffic, the scenario information, which has already been prepared, is transmitted to and stored on each drone before the flight, as shown in Figure 5. In addition, the correction data from the RTK-GPS base station are transmitted through broadcasting. Consequently, the amount of data transmitted from the GCS to the drones is constant, as follows:

(2) $$d_{s2}=d_{base}\times 1+n\times d_{status}$$

**Figure 6:**
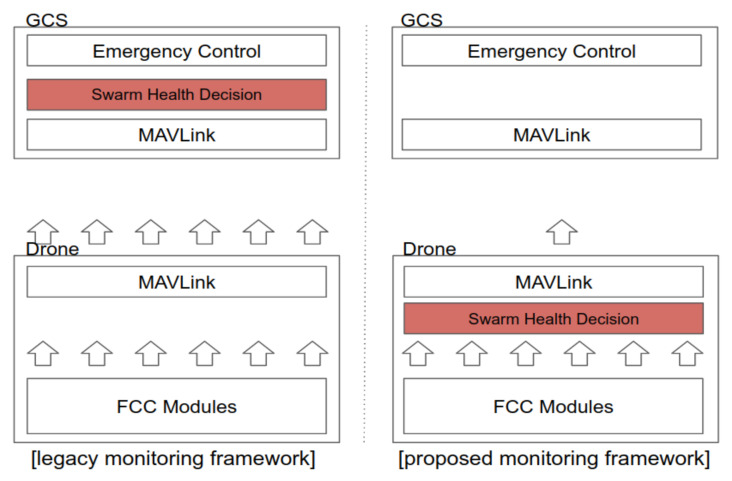
Comparison of monitoring methods.

However, if each drone needs to transmit several pieces of information, such as the sensor data for health status monitoring, the amount of data transmitted from the drones to the GCS will increase in proportion to the number of drones. To minimize the amount of data transmitted in this direction, in the proposed swarming flight system, the health status of each drone is not checked using the ground station system. Instead, the health status of each drone is checked at the drone by using a health decision module, as shown in Figure 6. The health decision module checks whether the drone is healthy in terms of each considered status indicator and expresses each status result as a bit. In addition, to further minimize the information sent from the drones to the ground station, this information is allowed to be transmitted only when changes occur.

**Figure 7:**
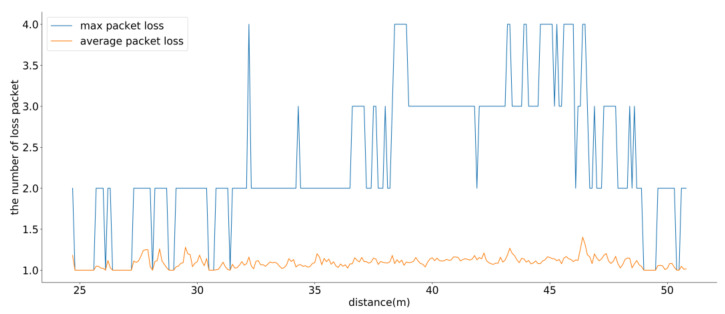
Results of the maximum and average burst packet loss.

To verify the proposed communication system, an experiment was conducted over a continuous period of 360 seconds based on a scenario with 100 drones. Figure 7 shows the maximum and average packet losses from the GCS to the drones for the RTK-GPS correction data as functions of the distance. In most cases, only one packet was dropped. In terms of the maximum packet loss, up to 4 packets were dropped. Therefore, the proposed system could maintain the fixed RTK-GPS mode.

### 2.3. Position Estimation

The proposed system utilizes RTK-GPS sensors in combination with inertial measurement units (IMU) and barometric sensors to realize accurate position estimation. In the RTK-GPS technique, measurements of the phase of the signal’s carrier wave are used in addition to the information content of the signal, and a base station or interpolated virtual station provides real-time corrections to achieve up to centimeter-level accuracy [24]. However, if the satellite signal is weak or the communication conditions are inferior, the precise signal may be missed, or the incorrect value may be reported. In a swarming flight system, this situation is undesirable because the drones are located close to one another and are likely to collide. To overcome this problem, a mode switching algorithm is proposed for the swarming flight system to change the sensor fusion mode depending on the RTK-GPS conditions. When the RTK-GPS conditions are inferior, the GPS mode, in which the GPS information is mainly used along with the IMU and barometer data, is applied. Once the RTK-GPS conditions improve, the mode is switched back from the GPS to RTK-GPS. The objective is for the RTK-GPS mode to be used as much as possible.

**Figure 8:**
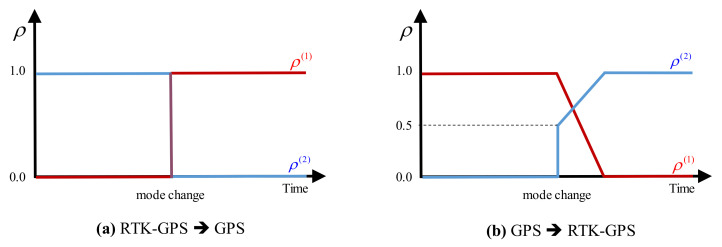
Smooth mode switching.

Notably, when the mode changes, especially from the GPS to RTK-GPS mode, several position errors may accumulate in the estimation due to the GPS sensor error. This phenomenon may lead to sudden position changes, which may cause the spectators of the drone show to feel insecure. To solve this problem, a modified position estimation algorithm is proposed to enable smooth prediction of the future positions. The proposed position estimation algorithm is designed to predict the position while smoothly varying the information ratio (ρ) when switching from the GPS information to the RTK-GPS information, as shown in Figure 8.

To implement this approach, the modified position estimation algorithm is applied to enhance the position estimator module of the PX4 FCC [37]. Using the position and velocity from sensors are measured, the position and velocity information are predicted through the accelerometer, as follows:

(3) $${\bar{p}}_{k}={\hat{v}}_{k-1}\Delta t+\frac{1}{2}a_{k-1}\Delta t^{2}+{\hat{p}}_{k-1}$$

(4) $${\bar{v}}_{k}=a_{k-1}\Delta t+{\hat{v}}_{k-1}$$

where ${\bar{p}}_{k}$ and ${\bar{v}}_{k}$ denote the predicted position and velocity at $k^{th}$ time. ${\hat{p}}_{k-1}$ and ${\hat{v}}_{k-1}$ indicate the corrected position and velocity. $a_{k-1}$ is the value of acceleration. The predicted value is compared with the information received from RTK-GPS and GPS sensor and each error is measured, as follows:

(5) $$e_{p,k}^{\left(i\right)}=z_{p,k}^{\left(i\right)}-{\bar{p}}_{k}$$

(6) $$e_{v,k}^{\left(i\right)}=z_{v,k}^{\left(i\right)}-{\bar{v}}_{k}$$

where $i^{th}$ is sensor number (1: GPS, 2: RTK-GPS). $e_{p,k}^{\left(i\right)}$ and $e_{v,k}^{\left(i\right)}$ indicate position and velocity error of $i^{th}$ sensor between measured and predicted value at $k^{th}$ time. $z_{p,k}^{\left(i\right)}$ and $z_{v,k}^{\left(i\right)}$ are measured position and velocity respectively. 

After that, the correction of the position and velocity vectors was made using the position and velocity errors in Equations (5) and (6), as follows

(7) $${\hat{p}}_{k}={\bar{p}}_{k}+\sum_{i}^{2}e_{p,k}^{\left(i\right)}w_{p}^{\left(i\right)}\rho^{\left(i\right)}\Delta t$$

(8) $${\hat{v}}_{k}={\bar{v}}_{k}+\sum_{i}^{2}e_{v,k}^{\left(i\right)}w_{v}^{\left(i\right)}\rho^{\left(i\right)}\Delta t$$

where $w_{p}^{\left(i\right)}$ and $w_{v}^{\left(i\right)}$ denote weight of position and velocity of $i^{th}$ sensor. 

Then, the accelerometer bias is updated with the error value, as follows:

(9) $$a_{k+1}=a_{k}-\sum_{i}R_{E}^{B}(e_{p,k}^{\left(i\right)}w_{p}^{\left(i\right)}{}^{2}+e_{v,k}^{\left(i\right)}w_{v}^{\left(i\right)})\rho^{\left(i\right)}\Delta t$$

where $R_{E}^{B}$ is a transformation matrix from global to body coordinate system. 

**Figure 9:**
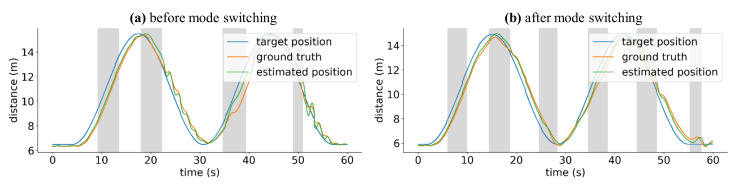
Mode switching results.

As a result of the mode switching algorithm, as shown in Figure 9, even if an error occurred due to a problem in the RTK-GPS position information, the affected drone will return smoothly to its trajectory.

## 3. Scenario Generation

A scenario for a swarm of drones includes the trajectory of each drone at any given time. A scenario is typically divided into several scenes to be displayed in the sky. When creating scenarios, the transitions between the scenes must be considered. To ensure a safe transition, the trajectory of each drone should be designed to avoid collisions. The sum of the movement distances of the trajectories should be minimized to ensure rapid transition. Moreover, the total movement distance of each drone should be similar to ensure energy efficiency. The proposed swarming flight system can ensure safe and energy-efficient scene transitions while minimizing the transition times between the scenes during the creation of a scenario. In addition, the system can land the drones safely even when several drones encounter difficulties.

### 3.1. Problem Statement

**Figure 10:**
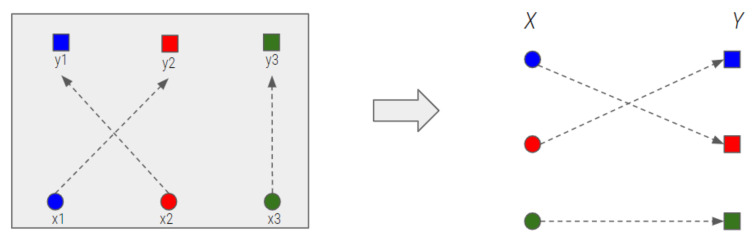
Scene transition (left: based on spatial coordinates, right: based on a bipartite graph).

To enable an efficient display, the scenes should transition as rapidly as possible. The problem of designing the drone movement for a scene transition is similar to an assignment problem in a bipartite graph whose vertices can be divided into two disjoint and independent sets X and Y such that every edge connects a vertex in X to an edge in Y, as shown in Figure 10 [38].

To represent the problem, let G=(X∪Y,E) be a bipartite graph, where X and Y represent the drones in the current scene and next scene respectively. $x_{i}$ denotes the $i^{th}$ vertex in X and $y_{j}$ denotes the $j^{th}$ vertex in Y, where $1\leq i\leq \left|X\right|$ and $1\leq j\leq \left|Y\right|$ [38,39]. $\left|X\right|$ and $\left|Y\right|$ denote the numbers of elements in X and Y, respectively. In this problem, $\left|X\right|$ and $\left|Y\right|$ are equal because the same number of drones is used throughout a scenario. $w(e_{x_{i}y_{j}})$ denotes nonnegative weight of $e_{x_{i}y_{j}}$ which is the edge connected between vertex $x_{i}$ and $y_{j}$. In this problem, the weight indicates the Euclidian distance between drone $x_{i}$ of the current scene X and drone $y_{j}$ of the next scene Y, as follows:

(10) $$w(e_{x_{i}y_{j}})=\sqrt{{\left(p_{x}^{x_{i}}-p_{x}^{y_{j}}\right)}^{2}+{\left(p_{y}^{x_{i}}-p_{y}^{y_{j}}\right)}^{2}+{\left(p_{z}^{x_{i}}-p_{z}^{y_{j}}\right)}^{2}}$$

where $\left(p_{x}^{x_{i}},p_{y}^{x_{i}},p_{z}^{x_{i}}\right)$ and $\left(p_{x}^{x_{i}},p_{y}^{x_{i}},p_{z}^{x_{i}}\right)$ the 3D position of drone $x_{i}$ and $y_{j}$, respectively.

All the edge weights in the graph are represented using a |X|×|Y| matrix C. This matrix denotes edge set in the complete bipartite graph whose vertices can be partitioned into two subsets, drones of current scene and next scene, as follows:

(11) $$C=\left[\begin{array}{ccc} w(e_{x_{1}y_{1}}) & \ldots & w(e_{x_{1}y_{n}})\\ \vdots & \ddots & \vdots \\ w(e_{x_{n}y_{1}}) & \ldots & w(e_{x_{n}y_{n}}) \end{array}\right]$$

Therefore, the given problem is to match $x_{i}$ to $y_{j}$ such that each vertex in X is connected to exactly one vertex in Y while minimizing the sum of the costs. In other words, the problem is to find a function σ(i), representing the index of a vertex, such that the sum of the cost functions is minimized, as follows:

(12) $$min\sum_{i=1}^{n}w_{x_{i}y_{\sigma (i)}}$$

### 3.2. Hungarian Algorithm

To find σ(i), the Hungarian algorithm, also known as the Kuhn-Munkres algorithm, is generally adopted [14,39,40,41]. This algorithm is an optimization method to solve the assignment problems to maximize or minimize a cost. In this paper, the Hungarian algorithm is used for cost minimization. We proceed to provide a brief description of the Hungarian algorithm to assist explaining the proposed algorithm. Algorithm 1 presents the Hungarian algorithm in the form of pseudocode.
**Algorithm 1. Pseudocode for the Hungarian algorithm Function Hungarian-Algorithm (**G**).** 
***% initial vertex feasible labeling***
$l_{1}$
 
i=1,j=1
 
$\forall x\in X,l_{1}(x)=\underset{x\in X}{\min}\{w(e_{xy})\}$
 
$\forall y\in Y,l_{1}(y)=0$
 
$M_{1}\leftarrow \;\mathrm{initial}\;\mathrm{matching}\;$
 
**while**
$\left|M_{i}\right|<\left|X\right|$
  
***% find the maximum matching***
M
  
$M_{i}^{j}=M_{i}$
  
**do**
   
$F_{i}^{j+1}\leftarrow \mathrm{alternating}\;\mathrm{forest}\;\mathrm{of}\;M_{i}^{j}\;\mathrm{rooted}\;\mathrm{at}\;\mathrm{the}\;\mathrm{unmatched}\;\mathrm{vertices}\;x\in X$
   
$P_{i}^{j+1}\leftarrow \;a\;\mathrm{path}\;\mathrm{of}\;F_{i}^{j+1}\;\mathrm{which}\;\mathrm{contain}\;\mathrm{another}\;\mathrm{unmatched}\;\mathrm{vertex}\;y\in Y$
   
$M_{i}^{j+1}=M_{i}^{j}⊕E(P_{i}^{j+1})$
   
$Q_{}^{j+1}=[X-V(F_{i}^{j+1})]\cup [Y\cap V(F_{i}^{j+1})]$
   
j=j+1
  
**while**
$\left|M_{i}^{j}\right|\neq \left|Q_{i}^{j}\right|$
  
$M_{i}=M_{i}^{j}$
  
***% update the feasible vertex labeling***
  
$F_{i}\leftarrow \mathrm{alternating}\;\mathrm{forest}\;\mathrm{of}\;M_{i}\;\mathrm{rooted}\;\mathrm{at}\;\mathrm{the}\;\mathrm{unmatched}\;\mathrm{vertices}\;x\in X$
  
$S=V(F_{i})\cap X$
  
$T=V(F_{i})\cap Y$
  
$\alpha =min_{x\in S,y\notin T}\{w(e_{xy})-l_{i}(x)-l_{i}(y)\}$
  
$l_{i+1}(v)=\left\{\begin{array}{c} l_{i}(v)+\alpha \\ l_{i}(v)-\alpha \\ l_{i}(v) \end{array}\right.\begin{array}{c} \mathrm{if}\;v\in S\\ \mathrm{if}\;v\in T\\ \mathrm{otherwise} \end{array}$
  
i=i+1
 
**done**


### 3.3. Proposed Algorithm

Although the Hungarian algorithm can find the minimum sum of the costs, an efficient transition between the scenes cannot be ensured because a scene transition is complete only once all drones move completely to the next scene position. Even when most drones have arrived at their destinations, the drones must wait until the last drone arrives because all the drones need to be deployed in their designated positions for the next scene. Consequently, the Hungarian algorithm is not efficient in terms of drone battery usage, and consequently, the overall operating time is reduced. Moreover, it is essential to minimize the maximum movement distance among the drones in a swarming flight scenario. In this paper, a new efficient assignment algorithm is proposed to minimize the maximum weight (distance) before the Hungarian algorithm is applied, as follows:

(13) $$\min\{\max_{x\in X,y\in Y}w(e_{xy})\}$$

To reduce the maximum weight, the cost matrix is updated by continuously removing the highest weight. However, an ideal matching may not be found in the bipartite graph if excessively many edges are removed. Therefore, the Hall theorem [42] is used to check that the perfect matching condition in a bipartite graph with $\left|X\right|=\left|Y\right|$ is satisfied. The bipartite graph G contains an ideal matching if and only if the Hall condition is satisfied, as follows:

(14) $$\left|S\right|\geq \left|N(S)\right|\;\mathrm{for}\;\mathrm{every}\;S\subseteq X$$

where N(S) denotes the neighbors of S and can be expressed, as follows:

(15) $$N(S)=\{y\in Y|e_{xy}\in E\;\mathrm{for}\;\mathrm{some}\;x\in S\;\}$$

In addition, the binary search algorithm, also known as half-interval search, is used to reduce the complexity. The binary search algorithm finds the position of a target value within a sorted set of weights W. This algorithm checks whether $E_{o}$ contains an ideal matching, according to the Hall theorem, up to the middle point m of the array. $E_{o}$ is the optimized set of edges in which all the edges having weights greater than the $m^{th}$ weight in the sorted weight set W have been removed, as follows:

(16) $$E_{o}=E-\{e_{xy}|w(e_{xy})\geq W_{m},x\in X,y\in Y\}$$

where $W_{m}$ denotes the $m^{th}$ component of W.

If $E_{o}$ contains an ideal matching, r is moved to m−1 to eliminate the edges with weights greater than the middle point of the weight set. If an ideal matching is not identified, l is moved to m to seek an ideal matching. This loop is repeated until l is the same as r. In this manner, the minimum sum of the distances is optimized while reducing the standard deviation of the distances. The pseudocode for this algorithm is presented in Algorithm 2.

Once assignment matching has been performed, the drones should be separated into several layers to avoid collisions while the drones are moving. Each layer is located in a different plane, which is tilted with respect to the ground, to establish separate movement paths for the transition between the scenes. To detect collisions between the drones, the intersections between the movement paths should be checked. In this paper, a movement path is defined by its start and end points and is treated simply as a line segment, i.e., we assume that each path is straight [43]. A line segment is a part of a line that is bounded by two distinct points, specifically, the start point ($p_{0}$) and end point ($p_{1}$). The points along the line segment can be represented in terms of u∈[0,1], as follows:

(17) $$p=p_{0}+u(p_{1}-p_{0})$$

To identify an intersection between two line segments l and m, the intersection point, where $p^{l}=p^{m}$, is calculated, as follows:

(18) $$p_{0}^{l}+u^{l}(p_{1}^{l}-p_{0}^{l})=p_{0}^{m}+u^{m}(p_{1}^{m}-p_{0}^{m})$$

Each point p can be expressed in terms of its coordinates (x,y) in a plane. Solving for the intersection point yields the following two equations in terms of two unknowns, $u^{l}$ and $u^{m}$, as follows:

(19) $$x_{0}^{l}+u^{l}(x_{1}^{l}-x_{0}^{l})=x_{0}^{m}+u^{m}(x_{1}^{m}-x_{0}^{m})$$

(20) $$y_{0}^{l}+u^{l}(y_{1}^{l}-y_{0}^{l})=y_{0}^{m}+u^{m}(y_{1}^{m}-y_{0}^{m})$$

**Algorithm 2. Pseudocode for the proposed Fair Hungarian algorithm** 
G=(X∪Y,E)
 $\begin{array}{l} W=\mathrm{sort}(\{w(e_{xy})|x\in X,y\in Y\}\\ l=1\\ r=\left|W\right|\\ \mathrm{while}\;l\neq r\\ \;m=ceil(\frac{l+r}{2})\\ \;E_{o}=E-\{e_{xy}|w(e_{xy})>W(m),x\in X,y\in Y\}\\ \;\mathrm{if}\left|S\right|\geq \left|N(S)\right|\;\mathrm{for}\;S\subseteq X\\ \;r=m-1\\ \;\mathrm{else}\\ \;l=m\\ \mathrm{done}\\ E_{o}=E-\{e_{xy}|w(e_{xy})>W(l),x\in X,y\in Y\} \end{array}$
 
$\hat{G}=(X\cup Y,E_{o})$
 
M
**= *Hungarian-Algorithm* (**
$\hat{G}$
**)**
 
L
**= Layer (**
M
**)**
$u^{l}$ and $u^{m}$ can be calculated, as follows:

(21) $$u^{l}=\frac{\left(x_{1}^{m}-x_{0}^{m})(y_{0}^{l}-y_{0}^{m})-(y_{1}^{m}-y_{0}^{m})(x_{0}^{l}-x_{0}^{m}\right)}{\left(y_{1}^{m}-y_{0}^{m})(x_{1}^{l}-x_{0}^{l})-(x_{1}^{m}-x_{0}^{m})(y_{1}^{l}-y_{0}^{l}\right)}$$

(22) $$u^{m}=\frac{\left(x_{1}^{l}-x_{0}^{l})(y_{0}^{l}-y_{0}^{m})-(y_{1}^{l}-y_{0}^{l})(x_{0}^{l}-x_{0}^{m}\right)}{\left(y_{1}^{m}-y_{0}^{m})(x_{1}^{l}-x_{0}^{l})-(x_{1}^{m}-x_{0}^{m})(y_{1}^{l}-y_{0}^{l}\right)}$$

If an intersection point between l and m exists, then $u^{l}$ and $u^{m}$ should satisfy the conditions $u^{l}\in [0,1]$ and $u^{m}\in [0,1]$ respectively.

However, even if no intersection exists between l and m, a collision may still occur in practice because of movement path errors or the influence of the finite drone size. Thus, a collision margin (ϵ) between l and m should be implemented.

Moreover, even if no intersection exists, a collision at the margin may occur at the start or end point. To detect a collision within the margin space, the minimum distance between the point and line is calculated at both $\left(l,p_{0}^{m}\right)$ and $\left(l,p_{1}^{m}\right)$.

In the case of $\left(l,p_{0}^{m}\right)$, $p_{0}^{m}$ is closest to the line at the tangent to l that passes through $p_{0}^{m}$. In other words, the dot product of the tangent and line is zero, as follows:

(23) $$(p_{0}^{m}-p^{l})\cdot (p_{1}^{l}-p_{0}^{l})=0$$

(24) $$(p_{0}^{m}-p_{0}^{l}-u^{l}(p_{1}^{l}-p_{0}^{l}))\cdot (p_{1}^{l}-p_{0}^{l})=0$$

(25) $$(p_{0}^{m}-p_{1}^{l})\cdot (p_{1}^{l}-p_{0}^{l})-u^{l}(p_{1}^{l}-p_{0}^{l})\cdot (p_{1}^{l}-p_{0}^{l})=0$$

(26) $$u^{l}=\frac{\left(p_{0}^{m}-p_{1}^{l})\cdot (p_{1}^{l}-p_{0}^{l}\right)}{\left(p_{1}^{l}-p_{0}^{l})\cdot (p_{1}^{l}-p_{0}^{l}\right)}$$

If $u^{l}<\epsilon$ when $u^{l}\in [0,1]$, a collision may occur.

To separate the layers, all the drone movement paths are iteratively checked for collisions. If no collision occurs among a set of paths, the paths can be included in the same layer.

**Figure 11:**
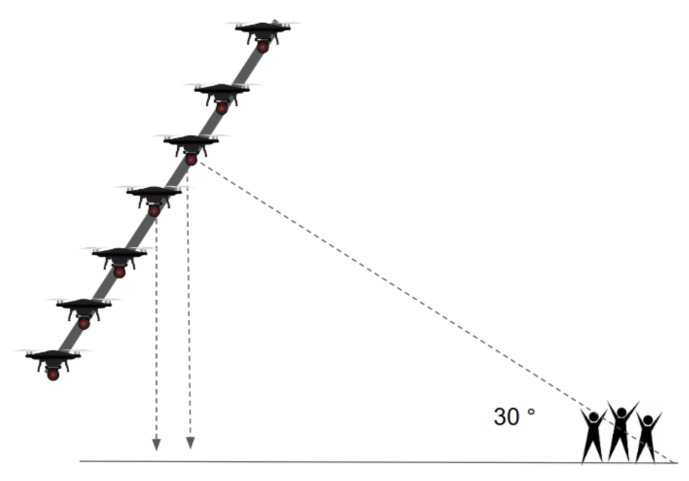
Proposed scene representation method.

Moreover, in the proposed scene representation method, the scene is tilted by approximately 30° for safe realization, as shown in Figure 11. This configuration can minimize the collisions with other drones when a drone falls or must land in an emergency situation. In addition, compared to the right-angle (vertical) view, the tilted view allows the desired pattern to be displayed more accurately with respect to the viewing angle of the spectators.

### 3.4. Numerical Example

**Figure 12:**
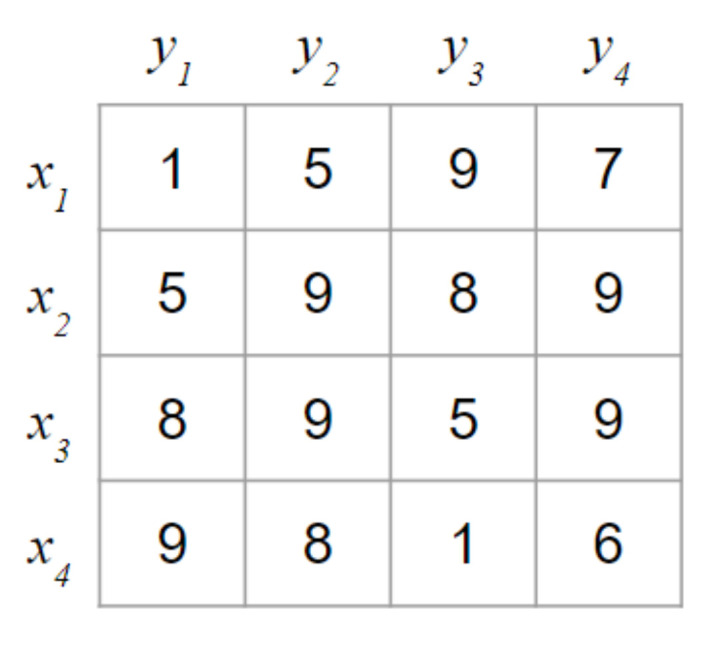
Example used for the numerical experiments.

Numerical experiments are conducted to illustrate the quality of the theoretical results and to compare the proposed Fair Hungarian algorithm with the original Hungarian algorithm. The numerical example focuses on the assignment problem. The numerical experiments were conducted by considering a scene transition involving 4 drones in each scene, as shown in Figure 12.

**Figure 13:**
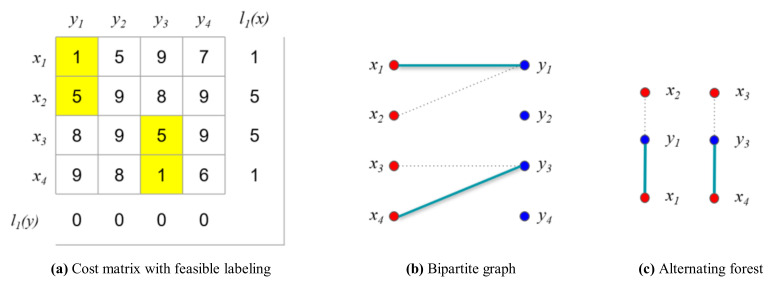
Example for the numerical experiment (*i* = 1, *j* = 1).

For the comparison, we first consider the original Hungarian algorithm presented in Algorithm 1. First, the initial vertex feasible labeling $l_{1}(x)$ is calculated with the minimum weights, as shown in Figure 13a.

Based on the initial vertex feasible labeling $l_{1}(x)$, the initial matching $M_{1}$ is greedily selected, as shown in Figure 13b. In the first iteration (i=1), no longest path P that contains another unmatched vertex y in the alternating forest exists that can be chosen to construct the maximum matching because no alternating path exists. Consequently, the size of the maximum matching is the same as that of the vertex cover Q, as follows:

(27) $$M_{1}^{1}=\{x_{1}y_{1},x_{4}y_{4}\},\;\left|M_{1}^{1}\right|=2$$

(28) $$Q=\{y_{1},y_{3}\},\;\left|Q\right|=2$$

**Figure 14:**
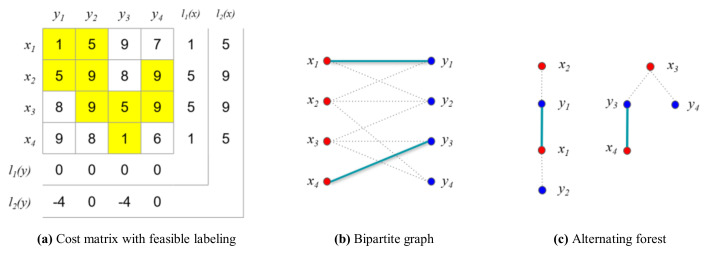
Example for the numerical experiment (*i* = 2, *j* = 1).

Once the maximum matching has been found, the feasible vertex labeling is updated using the alternating forest of $M_{1}$, as shown in Figure 13c. To find the vertex that will be updated for the next iteration, the sets S and T are identified based on the alternating forest. Subsequently, the feasible vertex labeling is updated using α, as shown in Figure 14a.

(29) $$S=\{x_{1},x_{2},x_{3},x_{4}\}$$

(30) $$V=\{y_{1},y_{3}\}$$

(31) $$\alpha =min_{x\in S,y\notin T}\{w(e_{xy})-l_{i}(x)-l_{i}(y)\}=4$$

In the second iteration (i=2), the matching $M_{2}$ is as shown in Figure 14b. The alternating forest based on the bipartite graph is shown in Figure 14c. The longest path in the forest is determined, as follows:

(32) $$P=x_{2}y_{1}x_{1}y_{2}$$

(33) $$E(P)=\{x_{2}y_{1},x_{1}y_{1},x_{1}y_{2}\}$$

(34) $$M_{2}^{1}=\{x_{1}y_{2},x_{2}y_{1},x_{4}y_{3}\},\;\left|M_{2}^{1}\right|=3$$

(35) $$Q=\{y_{1},y_{2},y_{3},y_{4}\},\;\left|Q\right|=4$$

**Figure 15:**
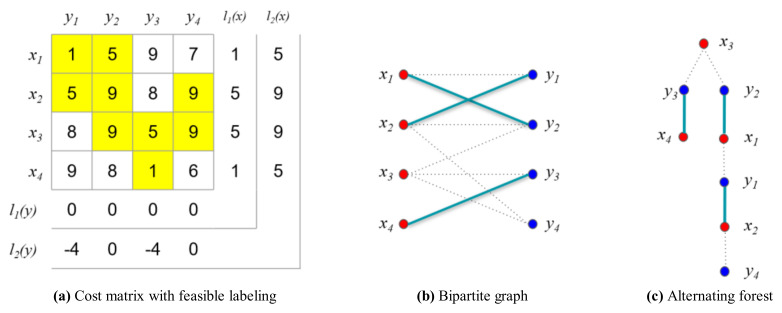
Example for the numerical experiment (*i* = 2, *j* = 2).

In this case, the matching is not an ideal matching because the number of edges in the matching, $\left|M_{2}^{1}\right|$, is not the same as in the vertex cover, $\left|Q\right|$. Therefore, the loop to determine the maximum matching must be iterated as shown in Figure 15.

(36) $$P=x_{3}y_{2}x_{1}y_{1}x_{2}y_{4}$$

(37) $$E(P)=\{x_{3}y_{2},x_{1}y_{2},x_{1}y_{1},x_{2}y_{4},x_{2}y_{1}\}$$

(38) $$M_{2}^{2}=\{x_{1}y_{1},x_{2}y_{4},x_{3}y_{2},x_{4}y_{3}\},\left|M_{2}^{2}\right|=4,\;$$

(39) $$Q=\{y_{1},y_{2},y_{3},y_{4}\},\left|Q\right|=4$$

**Figure 16:**
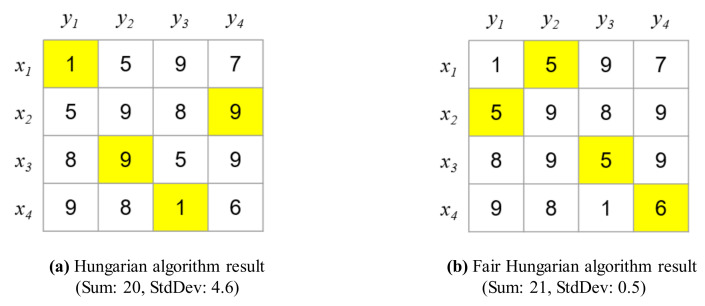
Comparison between the results of the Hungarian and Fair Hungarian algorithms.

Finally, the matching $M_{2}^{2}$ is an ideal matching because $M_{2}^{2}=\left|Q\right|=4$. Consequently, the total sum of the weights is 20. However, the variance is large, as shown in Figure 16a. In other words, certain drones consume considerably more energy than other drones, shortening the overall operating time.

In contrast, the proposed algorithm can reduce the variance, allowing the proposed system to achieve fair energy consumption and increasing the operating time of the drones as shown in Figure 16b. To reduce the variance, the cost matrix is first optimized in the proposed Fair Hungarian algorithm as presented in Algorithm 2. Before the cost matrix is updated, the weights are sorted, as follows:

(40) W={1,1,5,5,5,6,7,8,8,8,9,9,9,9,9,9}

Subsequently, the weights are removed by using the binary search algorithm until the Hall condition for the ideal matching is violated. Finally, the remaining weights are updated, as follows:

(41) W={1,1,5,5,5,6}

Once the weights are updated, the ideal matching M is identified using the Hungarian algorithm based on the cost matrix obtained after the removal of weights. The proposed algorithm reduces the variance to 0.5, even though the total sum of the weights is 21, which is similar to the optimized minimum cost, as shown in Figure 16b.

## 4. Results of a Swarming Flight Experiment

### 4.1. Implementation

#### 4.1.1. Drones

Since multiple drones are operated simultaneously in a swarming flight system, the drones should be constructed to be as simple as possible. Therefore, the proposed drone system is designed using simple modules, which are integrated in an interface board. The internal modules of each drone consist of an FCC module, an LED module, an RTK-GPS module, and a communication module.

**Figure 17:**
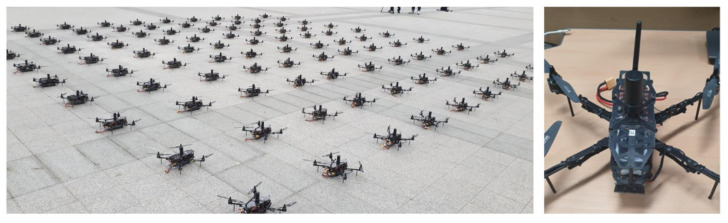
Swarming flight drones (left: deployed drones, right: swarming flight drone).

It is assumed that in the swarming flight operation, the scenario flight requires approximately 10 min, and 5 min are required for the automatic initial deployment and initial operation tests. Thus, the proposed drone as shown in Figure 17 is designed to fly for 15 min. In the comparison with the Intel drone, the speed and flight time of the proposed drone are approximately two times those of the Intel drone, and thus the proposed drone is more tolerant to wind and can support a longer demonstration time, as indicated in Table 2.

#### 4.1.2. Ground Control System (GCS)

**Figure 18:**
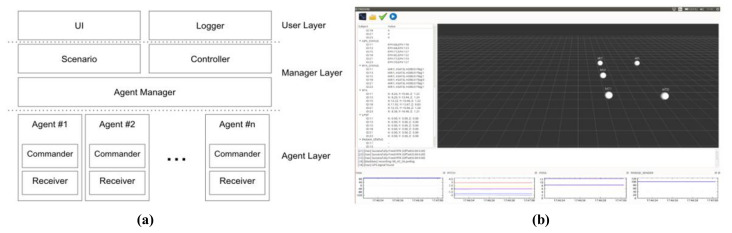
GCS architecture (**a**) and GUI (**b**).

For swarming flight operation, the GCS is implemented based on the Qt toolkit, which is an operating system independent framework, as shown in Figure 18. This system is designed to manage various heterogeneous drones to allow the use of different types of drones.

The architecture of the GCS is shown in Figure 18a. The system is divided into three layers: the user layer, manager layer, and agent layer. Each layer is independent of the other layers and communicates with the other layers by using a message queue. Consequently, a legacy layer can be easily replaced with a new layer. The user layer manages a graphical user interface (GUI). This system can illustrate the drones in 3-dimensional space in real time, as shown in Figure 18b. The control layer manages all the drones and implements the specified scenarios. In an emergency situation, the GCS can control each drone as required in the given layer. The agent layer manages the communication and sends commands to the drones. This agent module should be developed differently for different types of drones.

### 4.2. Experiment

To verify the proposed scene transition algorithm, a scenario consisting of 6 scenes was created. The scenes were named S1(‘init’), S2(‘3.1’), S3(‘100’), S4(‘flag’), S5(‘Korea’), and S6(‘KARI’). This scenario dataset is generated as an extensible markup language (XML) file and available at https://github.com/stmoon/swarm_flight_scenario (accessed on 23 January 2021). This scenario dataset includes the three-dimensional position (x,y,z) and yaw(φ) of each drone. Before operating the drone show, the drones are deployed as 10 × 10 like as shown in Figure 17a. Each drone which has a unique ID is initially placed 3m apart. With drone #1 as the origin, each drone has a relative position. For the experiment, a maximum of movement speed is limited by 4 m/s. The distance between drones in scenario was restricted to 1 m. The GCS is performed on a laptop computer with Intel(R) Core™ i7 CPU [45]. Each position of the drone is calculated using RTK-GPS. 

**Figure 19:**
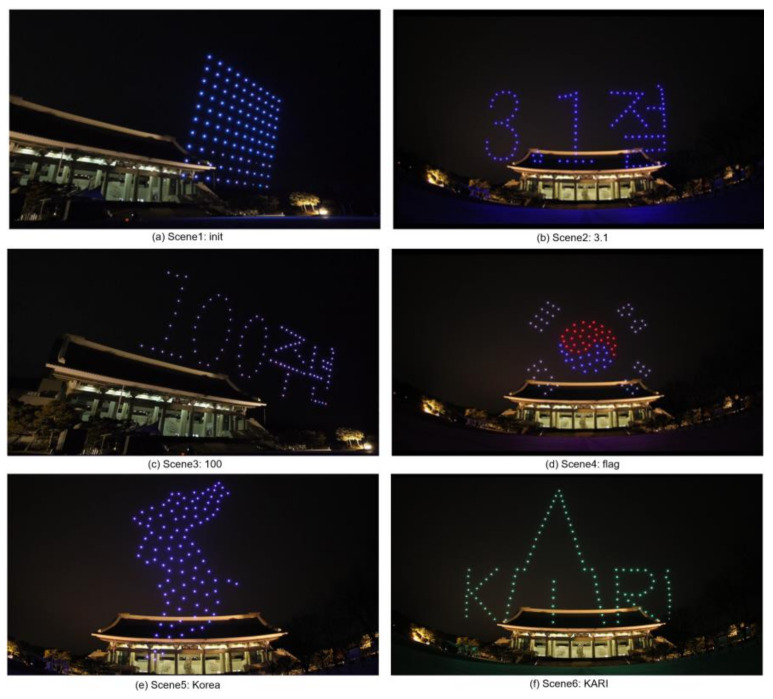
Drone show results.

To demonstrate the swarming flight formation, the drone show is operated at night as shown in Figure 19. It is performed during about 400 second for 6 scenes. The experimental result video is available at https://youtu.be/TgCKhgljWW8 (accessed on 23 January 2021).

### 4.3. Experimental Analysis

**Figure 20:**
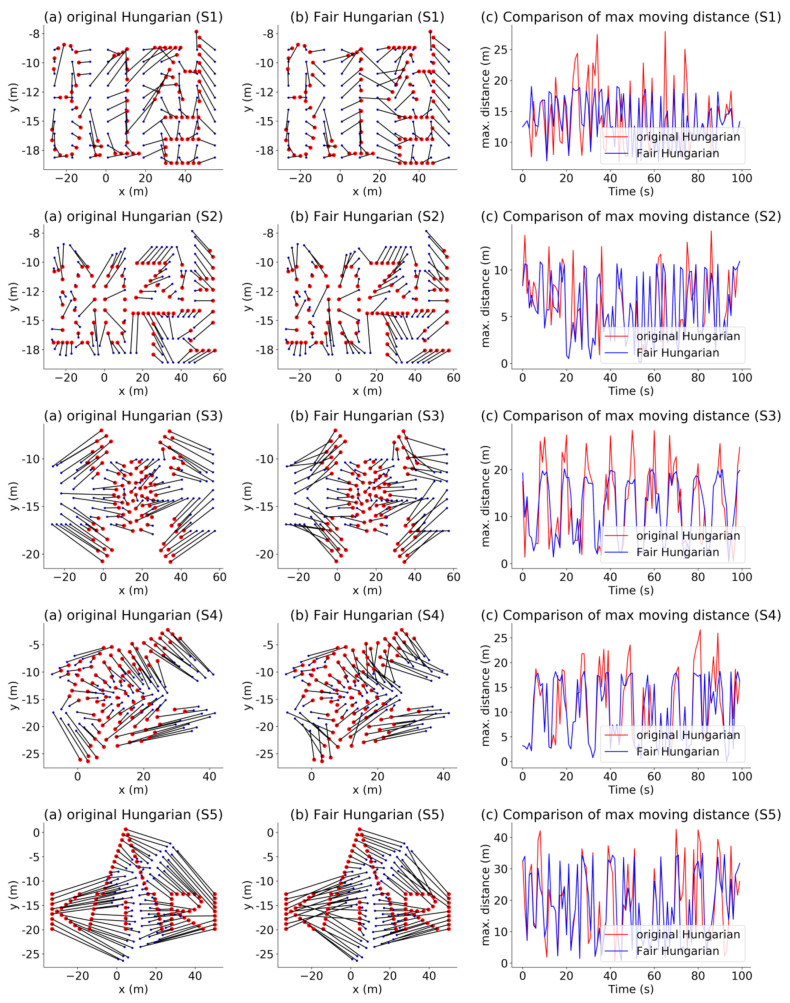
Scene transition.

To compare the proposed algorithm with the Hungarian method, Figure 20 shows the direction of movement of each drone during each scene transition pertaining to the original Hungarian algorithm (left panels) and the proposed algorithm (center panels) along with the graphs (right panels) comparing the maximum distance for each drone.

**Figure 21:**
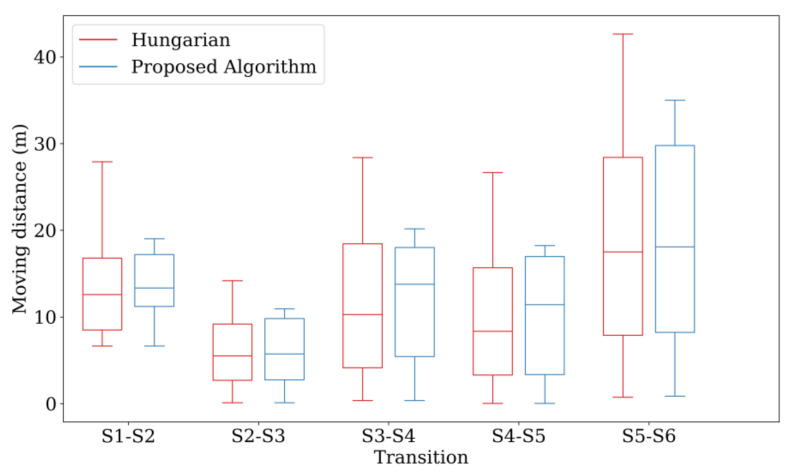
Results of the improved Hungarian method.

Figure 21 compares the sum of the distances, maximum movement distance among the drones, and standard deviation among the drones corresponding to the two Hungarian methods through boxplot. The proposed algorithm reduces the maximum movement distance among the drones, while the sum of the distances remains similar. In addition, the overall battery consumption of the drones is more efficient because the standard deviation is smaller than that achieved using the original Hungarian method. Consequently, even though the Hungarian algorithm can find the assignment with the minimum total cost, the energy consumption is not fairly distributed among all the drones. In contrast, the proposed algorithm can assign fair movement paths while maintaining a similar cost.

**Figure 22:**
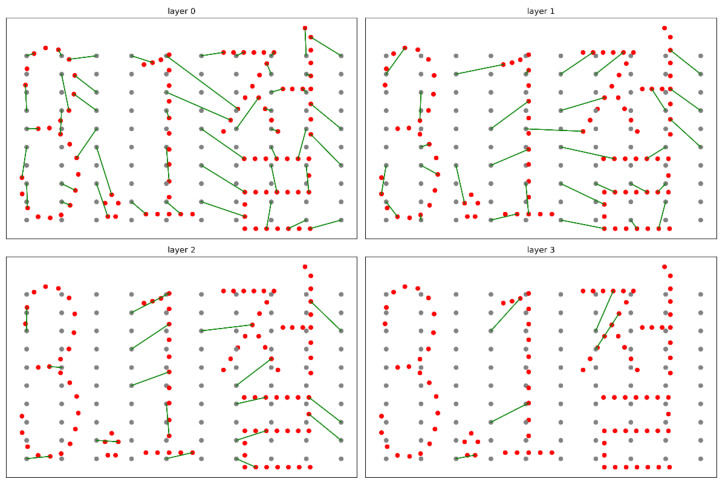
Layers for the second scene. (S1→S2).

In addition, each scene is separated into several layers to avoid collisions while the drones are moving. For example, in the case of the scene transition from S1 to S2, 4 layers are used, as shown in Figure 22.

## 5. Conclusions

For the efficient swarming flight formation transition of drone show, the Fair Hungarian algorithm is proposed to achieve fair energy consumption and thus increase the operating time of drones in swarm flight. The proposed algorithm equalizes the energy demand of drones by minimizing the maximum movement distance between drones in a swarming flight scenario. In addition, a robust and efficient swarming flight system is discussed including the method of efficient communication and robust position estimation. This algorithm and system have been verified through swarming flight experiments with 100 drones.

The swarming flight system proposed to date has been applied for artistic purposes. However, the system can be applied to scout and monitor the large areas within a short time. In this case, the Fair Hungarian algorithm will be used to extend the mission time by switching drones without collision.

## Figures and Tables

**Table 1:** Comparison with previous works.

	Application	Algorithm (σ(∗))	Weight (w)	Method	Verification (# of Targets)
Xiangming et al. [20]	Path planning	Original Hungarian	Quality	$max\sum_{i=1}^{n}w_{x_{i}y_{\sigma (i)}}$	Simulation (22)
Amir et al. [21]	Drone-station matching in Smart city	Original Hungarian	Energy	$max\sum_{i=1}^{n}w_{x_{i}y_{\sigma (i)}}$	Simulation (500)
Smriti et al. [19]	Multi-robot orchestra	Distributed Hungarian	Distance	$min\sum_{i=1}^{n}w_{x_{i}y_{\sigma (i)}}$	Demonstration (10)
Sarah et al. [22]	Fast and scalable task allocation	DHBA	Distance	$min\sum_{i=1}^{n}w_{x_{i}y_{\sigma (i)}}$	Simulation (50)
Our works	Scene transition in Drone show	Fair Hungarian	Distance	$\min\{\max_{i\in X,j\in Y}(w_{ij})\}$	Demonstration (100)

**Table 2:** Performance comparison of the Intel Shooting Star and the proposed drone.

	Shooting Star [44]	Proposed Drone (Figure 17)
Type	quadcopter	Quadcopter
Size	38.4 × 38.4 × 9.3 cm	50 × 41 × 16 cm
Weight	330 g	800 g
Flight time	5–8 min	10–15 min
Maximum speed	7 m/s	19.4 m/s
Rotor diameter	6 inches	8 inches

## Data Availability

The scenario data presented in this study are available at https://github.com/stmoon/swarm_flight_scenario (accessed on 23 January 2021).
